# When Access Becomes Fatal: A Case Series of Anesthetic-Drug Suicides Among Anesthesiologists

**DOI:** 10.7759/cureus.99036

**Published:** 2025-12-12

**Authors:** Naif Aljohani, Fahad Alenzi, Mohamed S Abdelrahman, Omar Aljohani, Yahya H Zakari, Faisal M Alzubaidi, Ibrahim A Almutairi, Basim M Alshahrani, Nourah Al Hogail, Abdulkreem Al-Juhani

**Affiliations:** 1 Forensic Medicine, Forensic Medicine Center, Riyadh, SAU; 2 Forensic Medicine, Ministry of Health, Riyadh, SAU; 3 College of Medicine, Imam Mohammad Ibn Saud Islamic University (IMSIU), Riyadh, SAU; 4 Forensic Pathology, Forensic Medicine Center, Riyadh, SAU; 5 Forensic Medicine, Forensic Medicine Center, Jeddah, SAU; 6 Surgery, King Abdulaziz University Faculty of Medicine, Jeddah, SAU

**Keywords:** anesthesiologist suicide, anesthetic drug misuse, fentanyl overdose, forensic toxicology, polysubstance intoxication, propofol toxicity

## Abstract

Anesthesiologists face a uniquely elevated risk of suicide, often through the misuse of anesthetic or centrally acting agents to which they have professional access. These deaths present substantial forensic challenges due to rapid drug metabolism, limited routine toxicology coverage, and the need for careful scene correlation. Our aim is to describe and compare the forensic, toxicological, and scene findings of three anesthesiologists who died by suicide through self-administration of anesthetic or centrally acting drugs and to highlight the implications for forensic investigation and prevention. This is a retrospective descriptive analysis study that was conducted using complete medicolegal autopsy files. Data included scene observations, external and internal examinations, radiologic findings, and comprehensive toxicology. Cases were synthesized into comparative tables to evaluate shared features, drug-selection patterns, and forensic signatures. The results showed that all three individuals died from drug-induced respiratory arrest following deliberate administration of anesthetic or sedative agents. Case 1 involved high-level propofol toxicity; case 2 demonstrated fentanyl with elevated morphine metabolites; and case 3 showed extreme multisubstance toxicity, including propofol, antidepressants, opioids, and adjunct pharmaceuticals. Scene findings were consistent with intentional preparation, and no traumatic injuries or natural disease were present. Toxicological interpretation required targeted analytical methods due to rapid postmortem drug redistribution and degradation.

So in conclusion, these cases illustrate the distinctive forensic characteristics of suicide among anesthesiologists, where drug access and expertise strongly shape the method of death. Accurate interpretation requires integrated scene analysis, targeted toxicology, and awareness of the unique pharmacologic challenges associated with anesthetic agents. Enhanced drug-security measures, early mental-health intervention, and vigilance within anesthesiology departments are essential to reduce the occurrence of similar fatalities.

## Introduction

Suicide among healthcare workers is a recognized occupational risk, with multiple meta-analyses indicating that physicians have elevated suicide rates relative to the general population [1]. Anesthesiologists constitute one of the most susceptible subsets within the medical workforce, with research consistently indicating a higher suicide rate compared to other specialties [2]. The heightened risk is associated with distinct occupational pressures, including high-intensity clinical settings, psychological stressors, and a culture of perfectionism, along with easy access to potent anesthetic agents that may unintentionally promote both impulsive and premeditated self-harm [1,2]. 

A notable characteristic of suicide among anesthesiologists is the utilization of anesthetic agents as the method of demise. Previous forensic analyses have shown deaths associated with substances including propofol, neuromuscular blockers, benzodiazepines, and combined sedative protocols, typically delivered intravenously by skilled professionals [3,4]. This approach is exceedingly rare outside of medical environments; almost all documented fatalities due to anesthetic overdoses involve healthcare practitioners with regular access to these agents [4,5]. Moreover, prior studies have indicated that the victim's profession frequently influences the method of suicide, with access to anesthetics significantly affecting the circumstances of the case [4]. Nonetheless, suicide from anesthetic overdoses is rather few, and documented instances highlight that even among healthcare professionals, anesthetic-related fatalities predominantly arise from accidental overdoses due to overuse rather than deliberate self-harm [4]. 

From a forensic perspective, such fatalities pose significant diagnostic difficulties. Numerous anesthetic agents deteriorate swiftly or are absent from normal toxicological screenings, complicating their identification. Agents such as atracurium decompose rapidly, resulting in the presence of only metabolites such as laudanosine in postmortem samples [5]. Previous case investigations indicate that specialist analytical procedures, such as GC-MS, may be necessary to verify the presence of anesthetic drugs in blood, adipose tissue, or hair [3]. Consequently, ascertaining the cause and manner of death frequently necessitates a multimodal strategy that incorporates scene examination, autopsy results, pharmacological knowledge, and specific toxicological analysis. 

Due to the infrequency and forensic intricacy of such situations, comprehensive recording is essential for enhancing knowledge in forensic medicine. This case series details three anesthesiologists who committed suicide utilizing anesthetic or centrally acting drugs, highlighting the variety of toxicological profiles, the nuances of scene interpretation, and the unique investigative issues these fatalities provide.

## Case presentation

Methodology 

This study utilized a retrospective case-series methodology to investigate three fatalities of anesthesiologists who committed suicide by the deliberate administration of anesthetic or centrally acting pharmaceutical drugs. Cases selected were from medicolegal autopsy records that had full forensic datasets accessible, encompassing scene reports, thorough exterior and internal examinations, radiologic evaluations, and extensive toxicological analysis. Deaths were recorded upon verification of a suicidal nature, lack of third-party involvement, and exclusion of natural disease processes. 

All autopsies were conducted in accordance with established forensic pathology protocols. The external inspection recorded postmortem alterations, indications of intervention, injection sites, and any traumatic injuries. An internal examination evaluated the principal organ systems to exclude structural pathology or hidden damage. Comprehensive radiography was systematically performed to assess skeletal integrity and identify hidden injuries or retained foreign objects. Toxicological analysis was conducted on postmortem blood, urine, vitreous humor, and organ specimens utilizing approved analytical techniques proficient in identifying anesthetic drugs, opioids, antidepressants, cannabis, alcohols, and other pertinent chemicals. 

Quantitative drug concentrations were analyzed in comparison to established therapeutic, toxic, and deadly reference ranges. Histopathological investigation was conducted as warranted by macroscopic observations. Scene investigation reports provide contextual details like body orientation, ambient disturbance, and the existence of paraphernalia linked to drug use. All forensic findings were methodically retrieved and synthesized. A comparative analysis was performed utilizing two structured result tables to outline common characteristics and discern new toxicological and behavioral trends among the three cases.

Case presentations

Case 1

A 40-year-old female anesthesiologist was found comatose in a clinical location where she had last been observed preparing for usual activities, with the scene remaining undisturbed and devoid of signs of external influence. She exhibited emaciation, with established posterior lividity and an absence of decomposition, accompanied by a distinct needle puncture mark on the dorsal aspect of her left hand. Her face, conjunctivae, and upper chest exhibited severe congestion accompanied by pronounced cyanosis of the lips and nails, along with dispersed petechial hemorrhages, characteristics indicative of fast respiratory failure. No traumatic injuries or defensive wounds were observed, and radiological imaging verified the integrity of the bones and the lack of foreign objects. Toxicological examination indicated significantly raised propofol levels, with blood concentrations at 363 ng/L and urine concentrations at 1030 ng/L, indicative of a high, intentionally provided dose well beyond therapeutic exposure and capable of inducing abrupt apnea. All other drug classes, such as alcohols, opioids, stimulants, sedatives, and illicit substances, yielded negative results, reinforcing the notion that propofol was the sole cause of the terminal event. The injection mark, elevated anesthetic concentration, tranquil environment, and absence of trauma all indicated that the cause of death was suicide acute respiratory failure resulting from propofol poisoning.

Case 2

A 41-year-old male anesthesiologist was discovered supine in his private office after colleagues noticed his extended absence. A minor contusion above his eyebrow suggested trauma during his collapse, and a nearby basin contained four syringes, three of which were used, indicating recent drug preparation. He seemed overweight externally and exhibited full rigor mortis and dark lividity throughout the body, along with prominent conjunctival and thoracic petechiae, significant facial congestion, and cyanotic lips and fingernails. Four needle puncture wounds were observed on the dorsum of the left hand, which was wrapped with a transparent covering, and there was bloody flow emanating from the nose and mouth. No indications of violence or resistance were apparent. Radiologic imaging verified the lack of fractures and foreign objects. Toxicology indicated significant fentanyl exposure, with blood levels at 0.28 ng/L and urine positive at 8.7 ng/L, quantities recognized as potentially deadly without breathing assistance. Furthermore, urine examination revealed significantly higher morphine levels at 8547 ng/L, corroborating substantial opioid consumption. The trace ethanol found in the blood was negligible and ascribed to postmortem phenomena, whereas all other examined drug categories, including stimulants, benzodiazepines, cocaine, cannabis, and barbiturates, yielded negative results. The existence of numerous syringes, elevated opioid concentrations, and the absence of severe injuries unequivocally indicate deliberate overdose. Death was consequently ascribed to suicidal acute respiratory failure resulting from high-level fentanyl poisoning, compounded by further morphine exposure.

Case 3

**Figure 1:**
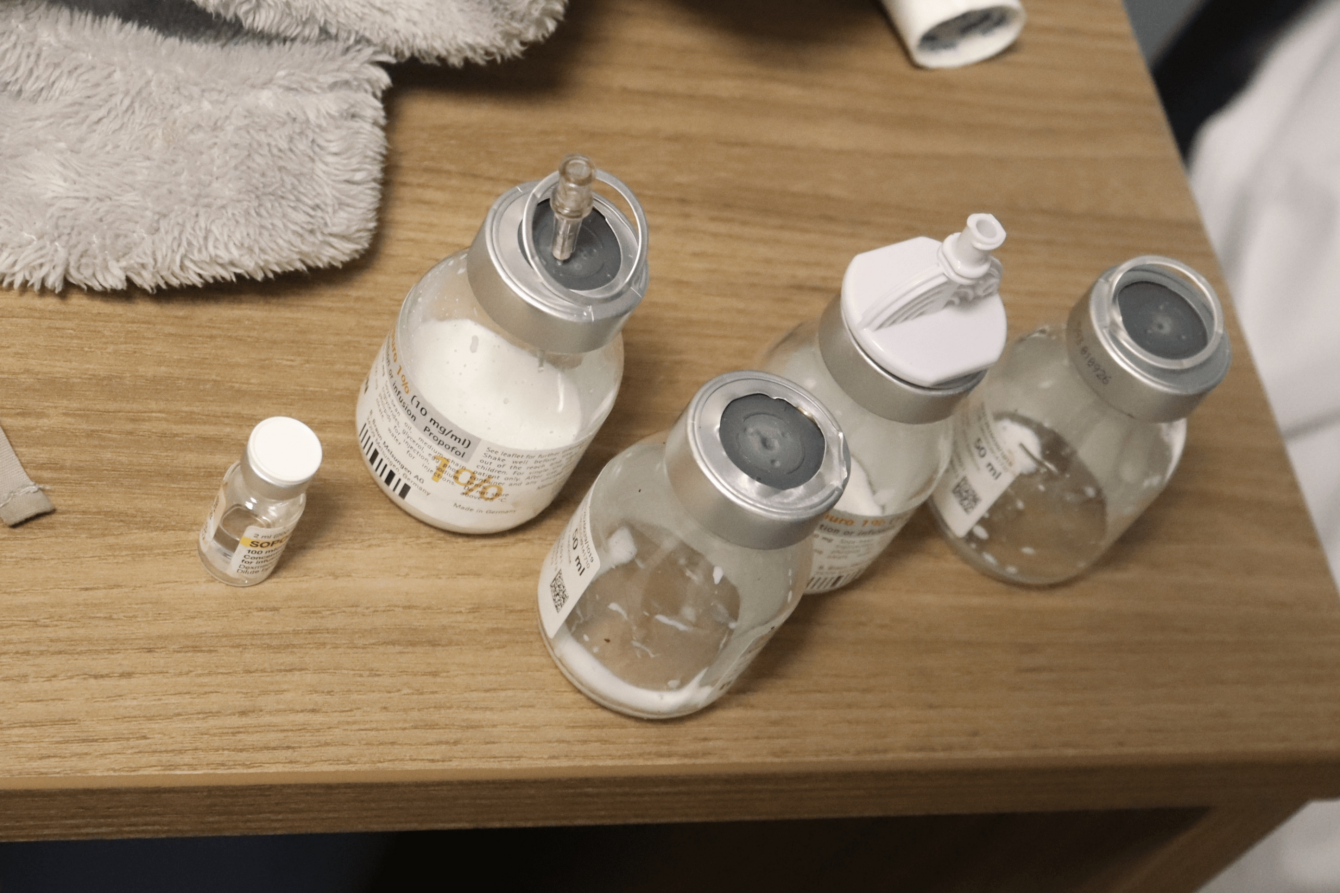
Anesthetic drug vials and preparation materials at the scene Photograph of the preparation trolley found beside the deceased anesthesiologist in case 3. The image shows multiple nearly empty 50 mL vials of 10 mg/mL propofol, used syringes, and associated drug-handling materials. These findings reflect deliberate preparation and administration of anesthetic agents prior to collapse

**Figure 2:**
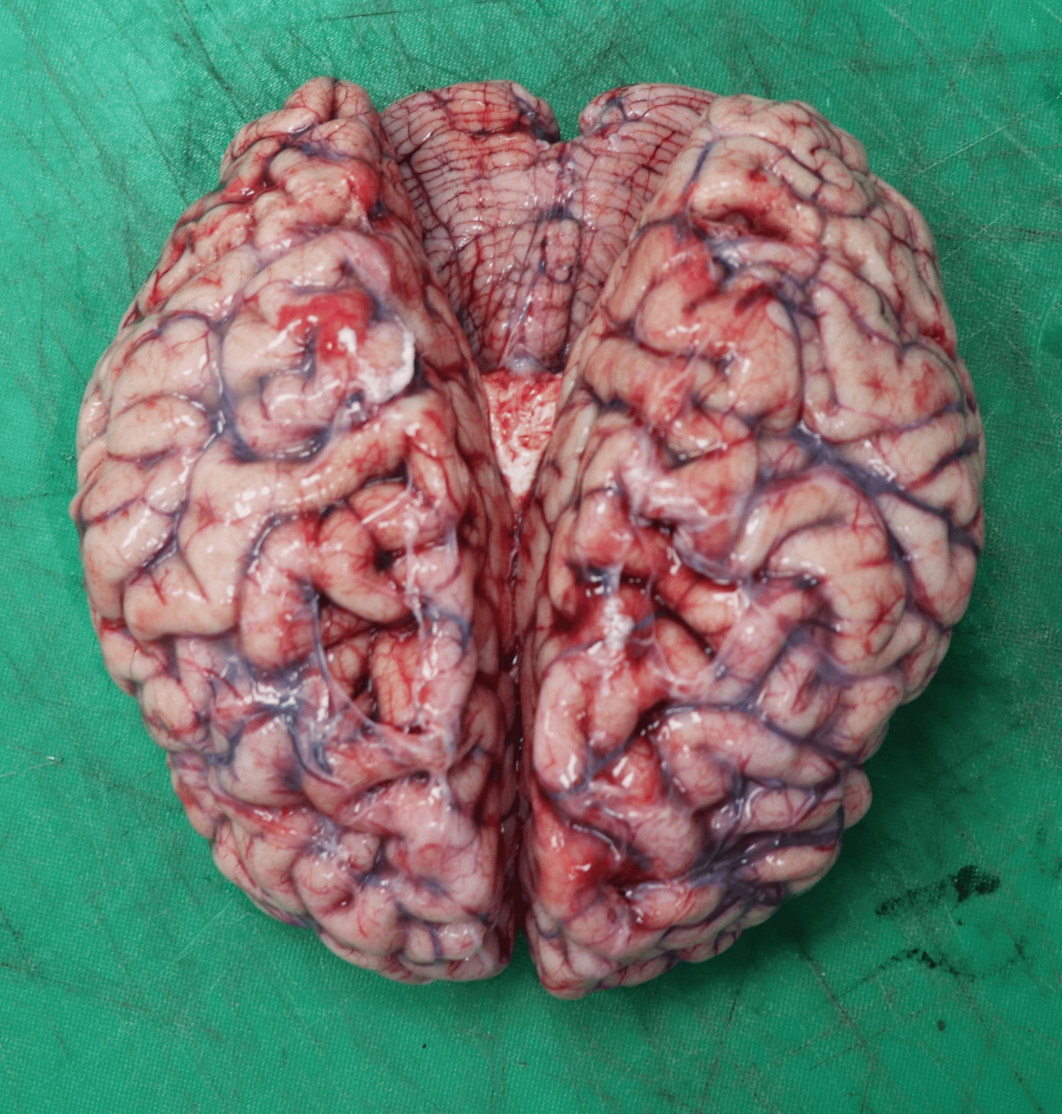
Congested brain of case 3 Gross examination of the brain demonstrating marked congestion and edema, consistent with hypoxic–ischemic injury secondary to combined anesthetic and CNS-depressant drug toxicity. The brain weighed 1300 g, indicating significant swelling

**Figure 3:**
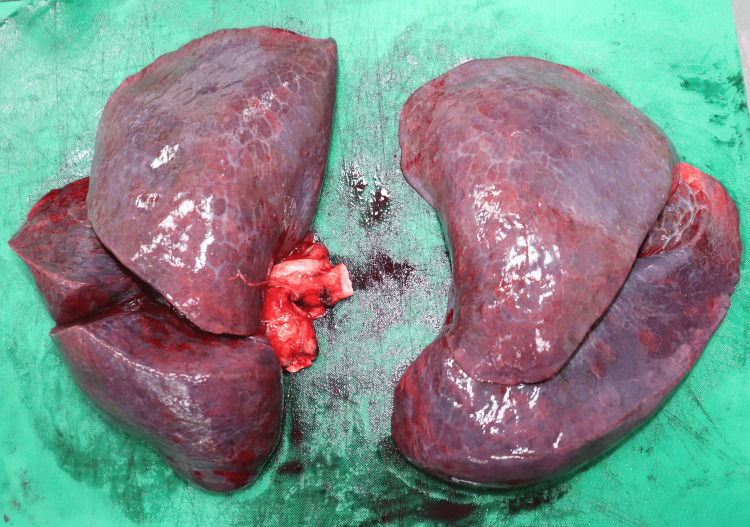
Congested and edematous lungs of case 3 Gross pathology image of the lungs showing severe congestion and edema. The right lung weighed 1146 g and the left lung, 1052 g, both markedly exceeding normal physiological weights. These findings are consistent with acute respiratory failure due to multi-substance overdose

A 31-year-old female anesthesiologist was discovered collapsed in a preparation room shortly before commencing her clinical responsibilities, adjacent to a trolley containing anesthetic agents (four almost empty 50 ml vials of 10 mg/ml propofol) (Figure 1), empty syringes, and assorted medications. The nearby surroundings exhibited no indications of conflict or unauthorized entry. She had an average physique, displayed early fixed lividity, and presented a subtle contusion above the left eyebrow indicative of impact with a proximate surface during a fall. Her attire remained unblemished and clean, with no defensive injuries or external harm observed. The inside examination revealed entirely normal organs except for a congested brain weighing 1300 g and congested, edematous lungs, with the right lung weighing 1146 g and the left lung weighing 1052 g. Figures 2-3 show the absence of fractures, no airway obstruction, and no natural condition that may account for the death. Toxicology indicated a significantly elevated multi-substance profile, characterized by exceptionally high propofol concentrations-24.2 µg/mL in blood and 1668.2 ng/mL in urine-levels that are much beyond anesthetic induction ranges and fall within recognized fatal thresholds. Furthermore, venlafaxine and norvenlafaxine were detected at concentrations of 17.24 µg/g and 6.9 µg/g, respectively, both substantially exceeding therapeutic expectations. The urine had significantly elevated levels of opioid metabolites, including codeine at 21,152 ng/g and morphine at 1,099 ng/mL, in addition to lidocaine, acetaminophen, and a cannabis metabolite (11-nor-Δ9-THC-COOH), indicating a multi-faceted polypharmacy exposure. The simultaneous presence of several potent CNS depressants, especially in elevated concentrations, alongside a nonviolent setting, unequivocally suggested deliberate self-administration. The fatality stemmed from suicidal combined drug toxicity involving propofol, opioids, antidepressants, and other sedative agents.

Results

Three deceased anesthesiologists were evaluated, all of whom died by suicide via the intentional self-administration of anesthetic or centrally acting agents. Notwithstanding commonalities in professional background and access to strong medications, each case had a unique toxicological and forensic pattern. Table 1 provides a succinct review of the principal demographic, scene, autopsy, and toxicology data, emphasizing the critical factors across the three instances and illustrating the fundamental forensic disparities that arose. 

**Table 1:** Summary of key findings across the three suicidal anesthesiologist cases

Parameter	Case 1	Case 2	Case 3
Age/sex	40/female	41/male	31/female
Discovery circumstances	Found unresponsive in the clinical area; scene calm and undisturbed	Found supine in the office; syringes nearby	Found collapsed in the preparation room, next to 4 almost empty vials of propofol
Scene indicators	Single injection mark; no disturbance	Four syringes (3 used); minimal blood at scene	Multiple anesthetic- and drug-containing vials and syringes present
External findings	Cyanosis, facial congestion, needle mark on left hand; no trauma	Marked petechiae, cyanosis, 4 injection marks; minor fall bruise	Mild eyebrow bruise, otherwise clean; no signs of struggle
Internal examination	No internal trauma; radiology negative	No internal trauma; radiology negative	Severely congested barin, lungs
Primary toxic agents	Propofol	Fentanyl + morphine	Propofol +venlafaxine/norvenlafaxine + opioids + lidocaine + cannabis
Toxicology level interpretation	High propofol concentration, consistent with deliberate overdose	High fentanyl level with elevated morphine, both in fatal ranges	Extremely high propofol, marked antidepressants, very high opioids, multi-substance burden
Co-detected substances	None	Trace ethanol (postmortem artifact)	Lidocaine, acetaminophen, cannabis metabolite
Manner of death	Suicide	Suicide	Suicide
Final pathophysiological mechanism	Acute respiratory failure from anesthetic overdose	Opioid-induced respiratory arrest	Combined CNS depression leading to fatal multi-system failure

In all instances, the remains were found in regulated healthcare settings, with no signs of exterior violence, defensive wounds, or forced access. The scene displays diversity, from a serene and untroubled collapse in Case 1 to the existence of numerous syringes in Case 2 and a comprehensively equipped anesthetic cart in Case 3. The external examination frequently revealed indicators of acute respiratory distress-cyanosis, petechiae, and facial congestion-though the severity and location of these findings varied between cases. Internal investigations of all three persons showed no traumatic lesions, natural diseases, or structural anomalies that could account for mortality, thereby corroborating a toxicological etiology in each case. Toxicology profiles varied significantly, indicating a range of overdose behaviors. 

Case 1 exhibited a solitary-agent overdose, characterized by significantly high propofol concentrations in both blood and urine, with no concomitant drugs found. Case 2 exhibited a dual-opioid profile, characterized by higher fentanyl concentrations and markedly increased morphine metabolites. Case 3 demonstrated the most pharmacologically intricate profile, with exceedingly elevated propofol levels in conjunction with venlafaxine, norvenlafaxine, morphine, codeine, lidocaine, acetaminophen, and a cannabis metabolite, constituting an atypical and concentrated amalgamation of CNS depressants. 

The disparities outlined in Table 1 highlight the personalized drug-selection practices exhibited by anesthesiologists with comparable access to banned medications. A comparative analytical study was conducted to better delineate the originality of these situations (Table 2).

**Table 2:** Comparative forensic patterns and novelty features among the three cases

Analytical domain	Case 1	Case 2	Case 3
Professional access pattern	Single-agent anesthetic misuse	Opioid-class anesthetic misuse	Multi-drug access (anesthetic + psychiatric + analgesic)
Substance selection behavior	Pure anesthetic overdose (propofol only)	Potent synthetic opioid + additional opioid metabolite	Complex polypharmacy reflecting layered toxicity intent
Drug complexity profile	Simple, single-peak pharmacology	Dual-opioid profile (fentanyl + morphine)	Highly complex CNS-depressant synergy (6+ agents)
Injection behavior	Single injection site	Multiple punctures (4 sites), structured preparation	Multi-route pattern implied (high levels + diverse agents)
Scene signature	Quiet, minimal disturbance	Syringe cluster and minor blood at the impact site	Active drug-handling environment with multiple vials
Physiological deterioration pattern	Rapid anesthetic-induced apnea	Opioid respiratory collapse with prominent petechiae	Progressive, multi-system CNS depression
Toxicological novelty	Isolated high-dose propofol fatality	Lethal fentanyl peak with elevated morphine	Exceptionally high propofol with antidepressants + opioids
Psychopharmacologic interpretation	Decisive single agent chosen for rapid effect	Use of powerful opioids consistent with clinical familiarity	Intentional stacking of depressants to ensure lethality
Forensic differentiating feature	Pure anesthetic suicide, no co-intoxication	Most pronounced asphyxial markers among cases	Most pharmacologically complex and uncommon combination
Overall novelty contribution	Rare single-agent propofol suicide	Distinctive fentanyl–morphine dual toxicity	Unusual, high-intensity multi-substance overdose in an anesthesiologist

This study illustrates that whereas each decedent had similar professional access to anesthetic medications, the patterns of misuse, substance complexity, and forensic signatures differed significantly. Case 1 utilized a rapid-acting anesthetic only, demonstrating a definitive single-agent approach. Case 2 employed a systematic, multi-injection opioid strategy, resulting in a notable syringe cluster and significant asphyxial indicators. Case 3 had deliberate pharmacologic stacking, integrating high-dose anesthetic drugs with antidepressants, opioids, and other medications, an atypical pattern in forensic literature. 

These distinctions underscore the variability of self-poisoning behaviors even within a specifically delineated professional cohort and enhance the originality of the current series. The collective findings from both tables highlight that, although all three individuals exhibited comparable occupational access and intentions, their drug selections, administration methods, scene characteristics, and toxicological intricacies were markedly different, providing novel insights into the spectrum of suicidal behaviors associated with anesthetic agents.

## Discussion

Overview of anesthesiologist suicide in forensic literature

The three cases demonstrate the unique susceptibility of anesthesiologists to suicide via medication poisoning, a phenomenon extensively documented in forensic and occupational literature. Initial investigations recorded the utilization of neuromuscular blockers like atracurium in self-inflicted fatalities, highlighting the impact of medication accessibility and pharmacological knowledge [5,6]. Subsequent cases demonstrated the common administration of combinations including propofol, benzodiazepines, and hypnotics to induce deep anesthesia prior to lethal respiratory failure [4]. Propofol has frequently been associated with anesthesiologist suicides, confusing interpretation due to postmortem redistribution. Recently, dual-agent suicides utilizing propofol and atracurium have been recorded among practitioners, reflecting the intricacy noted in the current series [3]. Extensive mortality studies indicate a heightened suicide prevalence among anesthesiologists relative to other physicians and the general populace [7], while comprehensive meta-analyses underscore workplace accessibility and stress as significant factors influencing suicide methods among healthcare professionals [1]. Systematic research pertaining to anesthesiologists indicates that poisoning, predominantly involving anesthetic drugs, is disproportionately utilized as a method of suicide within this field [2]. Regional studies similarly indicate elevated incidence of pharmaceutical approaches in physician suicides, often occurring within clinical settings [8]. Historical data further confirm that physicians generally select approaches necessitating medical expertise or regulated drug access, consistent with current cases [9].

Alignment with reported toxicological and behavioral patterns

Global forensic investigations have recognized anesthetic chemicals, particularly propofol, as prevalent factors in fatalities among anesthesia personnel, underscoring the worldwide significance of these incidents [10]. Monitoring programs in anesthesiology training indicate that systematic drug-screening techniques can significantly decrease substance consumption, implying a possible indirect reduction in suicide risk [11]. Recent professional standards have officially acknowledged suicide prevention as a priority in anesthesiology departments [12]. Patterns of diversion and misuse enhance contextual significance: propofol misuse is closely linked to insufficient drug security procedures within anesthesia facilities [13]. Historical mortality analyses indicate a sustained increase in suicide risk among anesthesiologists over several decades [14], whereas prospective surveys reveal drug-related occurrences as a frequent cause of mortality [15]. Thorough analyses ascribe these developments to a confluence of work stress, availability of fatal medications, and psychiatric risk factors [16]. Consistent with our findings, other case reports document suicides via neuromuscular blocking drugs like vecuronium, demonstrating analogous ways of self-induced respiratory arrest [17]. Additional accounts detail combinations of sedatives, neuromuscular blockers, and alcohol (e.g., etomidate with vecuronium), reflecting the polypharmacy trend observed notably in case 3 [13]. The alignment of these documented cases with the present series highlights the uniformity of forensic markers in anesthesiologist suicides: pharmacological accuracy, tranquil settings, fast-acting medicines, and polypharmacy approaches intended to guarantee fatality.

Forensic and toxicological challenges 

These instances exemplify several diagnostic challenges frequently associated with anesthetic-related fatalities. Many anesthetic drugs disintegrate rapidly postmortem or redistribute broadly, complicating interpretation even when quantitative levels appear low. The lipophilicity and fast dispersion of propofol can reduce postmortem blood concentrations even after a lethal dose, rendering scene correlation crucial [6]. Neuromuscular blockers, such as atracurium, are subject to fast hydrolysis, necessitating the precise identification of metabolites such as laudanosine to ascertain exposure [3,5]. Standard toxicology panels frequently omit anesthetic drugs, necessitating extensive analysis via gas chromatography-mass spectrometry (GC-MS) and liquid chromatography-tandem mass spectrometry (LC-MS/MS) in suspected instances [10]. Scene analysis is essential in differentiating suicide from accident or homicide: multiple agent preparation, syringe arrays, IV cannulation, and seclusion indicate intentionality; however, the lack of exertion marks or defensive injuries reinforces the determination of self-administration [17]. The polypharmacy pattern evident in case 3 underscores a further challenge, ascertaining whether the fatal mechanism resulted from a singular predominant agent or from synergistic CNS depression among many medications. Ultimately, interpretation must encompass autopsy results, toxicological analysis, professional insights, scene evidence, and psychosocial context.

Implications for prevention and professional monitoring

The forensic patterns observed in these cases carry important implications for prevention. Guidelines now recommend structured suicide-prevention frameworks within anesthesiology, including early identification of distressed clinicians, designated wellness leads, and improved reporting of physician suicides [12].

Institutional surveillance for drug diversion, clearer drug-access protocols, and tighter control of anesthetic agents, particularly propofol, are critical given the documented correlation between poor drug security and anesthetic misuse fatalities [13]. Drug-screening programs, such as long-term random urine testing in anesthesia trainees, demonstrated a complete elimination of substance-use incidents, representing a potential model for broader implementation [11].

Psychological support infrastructures remain equally essential. The high relapse rate among providers recovering from substance misuse [16] underscores the need for ongoing monitoring, compassionate intervention, and structured return-to-work pathways. Together, these preventive strategies emphasize that anesthesiologist suicides are not inevitable; they are preventable through integrated forensic awareness, occupational vigilance, and mental-health prioritization [18].

## Conclusions

This case series highlights the distinct forensic profile of suicide among anesthesiologists, characterized by deliberate use of anesthetics and centrally acting agents to produce rapid respiratory arrest. Despite differences in drug combinations and scene features, all cases demonstrated the same underlying mechanism: professional knowledge and access facilitating a precise, highly lethal method of self-harm. The toxicological complexity and rapid metabolic profiles of these agents underscore the importance of targeted analytical approaches in postmortem investigation. These findings reinforce the need for strengthened drug-security measures, proactive mental-health support, and early identification of distress within the anesthesiology workforce. Ultimately, recognizing the unique forensic signatures associated with these deaths can aid both accurate case interpretation and the development of preventive strategies in this high-risk specialty.

## References

[1] Dutheil F, Aubert C, Pereira B (2019). Suicide among physicians and health-care workers: a systematic review and meta-analysis. PLoS One.

[2] Bogod DG, McCombe K (2021). When worlds collide: territorial disputes and patient autonomy. Anaesthesia.

[3] Kort I, Hmandi O, Bekir O (2022). Sudden death due to a massive hydatid pulmonary embolism secondary to a cardiac cyst rupture. J Forensic Sci.

[4] Colucci AP, Gagliano-Candela R, Aventaggiato L, De Donno A, Leonardi S, Strisciullo G, Introna F (2013). Suicide by self-administration of a drug mixture (propofol, midazolam, and zolpidem) in an anesthesiologist: the first case report in Italy. J Forensic Sci.

[5] Martínez MA, Ballesteros S, Almarza E (2006). Anesthesiologist suicide with atracurium. J Anal Toxicol.

[6] Stoykova S, Kiryakova T, Nikolov D, Nedzhib A, Pantcheva I, Atanasov V (2018). Self-administered propofol-a case report of a physician suicide. Toxicol Anal Clin.

[7] Kopman AF, Checkoway H, Nagahama SI, Domino KB (2000). Cause-specific mortality risks of anesthesiologists. Anesthesiology.

[8] Hikiji W, Fukunaga T (2014). Suicide of physicians in the special wards of Tokyo metropolitan area. J Forensic Leg Med.

[9] Fitzsimons MG, Baker K, Malhotra R, Gottlieb A, Lowenstein E, Zapol WM (2018). Reducing the incidence of substance use disorders in anesthesiology residents: 13 years of comprehensive urine drug screening. Anesthesiology.

[10] Shinde S, Yentis SM, Asanati K (2020). Guidelines on suicide amongst anaesthetists 2019. Anaesthesia.

[11] Wischmeyer PE, Johnson BR, Wilson JE (2007). A survey of propofol abuse in academic anesthesia programs. Anesth Analg.

[12] Neil HA, Fairer JG, Coleman MP, Thurston A, Vessey MP (1987). Mortality among male anaesthetists in the United Kingdom, 1957-83. Br Med J (Clin Res Ed).

[13] Bruce DL, Eide KA, Smith NJ, Seltzer F, Dykes MH (1974). A prospective survey of anesthesiologist mortality, 1967-1971. Anesthesiology.

[14] Swanson SP, Roberts LJ, Chapman MD (2003). Are anaesthetists prone to suicide? A review of rates and risk factors. Anaesth Intensive Care.

[15] Ingale D, Ma B, Mugadlimath AB, Raju S, Jatti VB (2013). Suicide by intravenous injection of vecuronium bromide. J Indian Soc Toxicol.

[16] Detweiler CJ, Mambo NC (2014). Suicide with vecuronium and etomidate: a case report and review of the literature. Acad Forensic Pathol.

[17] Deng L, Wu L, Gao R, Xu X, Chen C, Liu J (2023). Non-opioid anesthetics addiction: a review of current situation and mechanism. Brain Sci.

[18] Mendelevich VD, Zalmunin KY (2015). Paradoxes of evidence in Russian addiction medicine. Int J Risk Saf Med.

